# Epidemiology characteristics of the drinking patterns and alcohol consumption among adults in Hainan Province, China

**DOI:** 10.3389/fpubh.2025.1490439

**Published:** 2025-03-17

**Authors:** Tingting Ou, Juan Jiang, Dingwei Sun, Ying Liu, Changfu Xiong, Xiaohuan Wang, Xue Zhou, Hongying Wu, Lijie Zhang, Chao Wang, Bin He

**Affiliations:** ^1^Institute for Tropical and Uncommunicated Disease Control and Prevention, Hainan Provincial Center for Disease Control and Prevention (Hainan Academy of Preventive Medicine), Haikou, Hainan, China; ^2^Hainan Provincial Center for Disease Control and Prevention (Hainan Academy of Preventive Medicine), Haikou, Hainan, China; ^3^Chinese Field Epidemiology Training Program, China CDC, CFETP, Beijing, China; ^4^Beijing Jishuitan Hospital, Capital Medical University, Beijing, China

**Keywords:** drinking pattern, alcohol consumption, adult age, Chinese population, public health

## Abstract

**Background:**

Alcohol consumption is a significant risk factor for premature mortality and increased disease burden worldwide, especially among young and middle-aged individuals. This study aims to evaluate drinking patterns and alcohol consumption among adults in Hainan Province, while also identifying associated factors.

**Methods:**

Analyses based on the 2022 “2 + 3” epidemiological survey in Hainan were conducted, and the drinking types, quantities, and frequencies among local residents were described. Chi-square tests and multiple linear regression were employed for the statistical analysis.

**Results:**

A total of 32,857 adults participated, yielding an overall drinking rate of 42.8%. The drinking rate was significantly higher among men (64.4%) than women (18.9%). The highest drinking rates were found in the 30–59 age group, especially among individuals aged 30–39. Ethnic minorities had a higher drinking rate (70.1%) than Han individuals. Lower educational attainment was associated with lower drinking rates, although the prevalence of active drinkers was higher. Men preferred strong liquor and beer, whereas women favored beer and rice wine. The average weekly alcohol consumption was 59.8 mL for men and 10.9 mL for women, with 43.6% of men exceeding 100 mL weekly, compared to 12.7% of women.

**Conclusion:**

This study emphasizes the complexity and diversity of drinking behaviors among adults in Hainan Province. Sociodemographic factors, including gender, age, ethnicity, education, marital status, occupation, and region, are closely linked to drinking behaviors. The findings provide a scientific basis for developing targeted public health strategies, highlighting the need for effective interventions to mitigate alcohol-related health issues among high-risk populations.

## Background

Alcohol consumption is a well-established risk factor for premature mortality and increased disease burden, especially among young and middle-aged populations. It surpasses other factors such as high body mass index, hypertension, and dietary risks (1–3). According to the World Health Organization’s 2024 report, approximately 400 million individuals worldwide have alcohol use disorders, with 209 million suffering from alcohol dependence. This situation led to about 2.6 million alcohol-related deaths in 2019 (4). The International Agency for Research on Cancer’s 2020 report estimates that approximately 741,000 new cancer cases globally are attributable to alcohol, accounting for 4.1% of all new cases that year. Notably, three-quarters of these alcohol-related cases occurred in men (5). Drinking behavior has been a significant focus in public health and social management, especially among young and middle-aged men. Their high drinking frequency and alcohol intake considerably impact health and social functioning (6, 7).

Approximately 1.6 million deaths worldwide each year are attributed to non-communicable diseases related to alcohol. This includes about 474,000 deaths from cardiovascular diseases and 401,000 from cancer. Additionally, around 724,000 deaths are due to injuries, such as traffic accidents and self-harm (4). Furthermore, alcohol suppresses immune function, increasing the risk of infectious diseases. In 2019, approximately 284,000 deaths globally were linked to alcohol-related infections (4). To effectively address the substantial disease burden caused by alcohol, the World Health Organization has launched the “2022–2030 Global Alcohol Action Plan.” The goal is to reduce harmful drinking by 20% compared to 2010 levels (8). While global alcohol consumption trends downward, China’s consumption continues to rise, with a 76% increase from 2005 to 2016. However, the abstinence rate has decreased from 50.9 to 42.1% (8). Therefore, strict control of alcohol consumption in China remains crucial.

Alcohol consumption exhibits notable demographic characteristics. Worldwide, drinking rates and alcohol-related health issues are generally higher among men than women, with episodic binge drinking being more prevalent (9). Distinct differences in drinking patterns exist across gender, age, geographic regions, and economic groups, highlighting the need for targeted alcohol consumption management. Currently, there is a lack of clear reporting on drinking types, quantities, and patterns among various demographic groups in China. This study aims to systematically assess alcohol consumption among adults in Hainan Province, highlighting differences in drinking patterns and alcohol intake across various demographic characteristics. The findings will provide a scientific basis for public health policy development, assist in identifying high-risk populations, and promote more refined and personalized intervention strategies.

## Methods

Data for this study were obtained from the 2022 “2 + 3” epidemiological sampling survey in Hainan Province, focusing on local residents and migrant populations to analyze drinking types, quantities, and corresponding alcohol consumption. In order to accurately understand the epidemic situation of hypertension, diabetes, tuberculosis, viral hepatitis (hepatitis Z and hepatitis C) and serious mental disorders (referred to as “2 + 3” diseases) and behavioral factors influencing public health in Hainan Province, the local government conducted the epidemic investigation. The present study aims to report the drinking patterns and alcohol consumption across demographic characteristics, providing a scientific basis for public health policy and facilitating the identification of high-risk populations.

### Study population and sampling

The study included residents aged 18 and older from Hainan Province, gathering demographic information such as gender (male, female), age, ethnicity (Han, minority), education level (primary or below, junior high, senior high, college or above), marital status (married, unmarried), occupation (agriculture, employed, student, other/unemployed), and geographic area (urban, rural). The sampling method for this survey is a two-stage unequal proportion cluster sampling. All of the village committees in cities, counties, and districts in Hainan Province were numbered and randomly selected weighted by the local population.

### Alcohol consumption data

#### Drinking rate

We assessed whether participants consumed alcohol in the past 12 months.

#### Types of alcohol

Participants consumed beer, high-proof liquor, low-proof liquor, wine, and yellow rice wine.

#### Average drinking quantity

Average consumption per drinking time was collected, measured in “liang” (50 mL) for all types except beer, which was measured in milliliters.

#### Drinking frequency

Past 12-month drinking frequency was documented and categorized as daily, 5–6 days per week, 3–4 days per week, 1–2 days per week, 1–3 days per month, or less than once per month. Drinking frequency was defined as follows: drinking on ≥5 days per week was classified as “active,” 1–4 days per week as “moderately active,” and ≤ 3 days per month as “inactive.”

#### Weekly pure alcohol intake

The average alcohol content was estimated for each type: beer (4%), high-proof liquor (50%), low-proof liquor (35%), wine (15%), and yellow rice wine (18%). Weekly pure alcohol intake was calculated using the formula: alcohol type content × drinking frequency × average drinking quantity, reported in milliliters.

### Statistical analysis

Chi-square tests were used to perform group comparisons for categorical variables, including drinking rates. Weekly alcohol intake was estimated based on average drinking quantity, types of alcohol consumed, and drinking frequency. The natural logarithm and exponential of the average weekly intake were calculated according to its distribution characteristics. Multiple linear regression and multivariable logistic regression analyses were conducted to assess the impact of various demographic characteristics on weekly alcohol intake. A significance level of *α* = 0.05 was employed, and data were analyzed using R version 4.3.2.

## Results

### Demographic information and overall drinking rate

**Figure 1:**
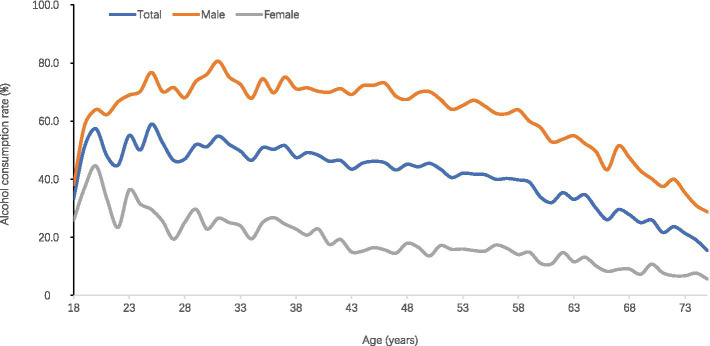
Alcohol consumption rate by age group.

This study included 32,857 adults from Hainan Province, of whom 17,733 (54.0%) were female. The overall drinking rate was 41.4%, with a weighted rate of 42.8%. The drinking rates were 64.4% for males and 18.9% for females. Among various age groups, adults aged 30–59 exhibited higher drinking rates, peaking at 50.3% in the 30–39 age group. Overall, the drinking rate initially increased before gradually declining, peaking at age 31 for males. In contrast, females experienced a brief rise from ages 18 to 20 before a decline (Figure 1). The drinking rate among ethnic minorities was significantly higher at 70.1%, compared to 36.8% for Han individuals. The majority of the population had a junior high education or less, with the lowest drinking rate of 31.3% observed among those with primary education or below, which was significantly lower than rates for higher educational levels. The drinking rate for married individuals was 40.4%, significantly lower than the 52.3% rate for unmarried individuals. The largest percentage of participants were farmers (52.3%), with a weighted drinking rate of 45.0%, which was lower than that of employed individuals at 51.7%. Drinking rates for urban and rural residents were similar, at 42.6 and 42.9%, respectively (Table 1). The highest drinking rates were found to be over 80% among male ethnic minorities aged 18–59 years. Drinking rates decreased from 21.8 to 3.7% with ages increased among Han female population, while around 50% ethnic minorities females drink at all age stages.

**Table 1:** Demographic information and drinking rates of participants.

Characteristics		N	%^cd^	Drinking rates		
				N	%^cd^	%^wtd^
Gender	Male	15,124	46.0	9,593	63.4	64.4^**^
	Female	17,733	54.0	3,994	22.5	18.9
Age (years)	18–29	2,263	6.9	1,190	52.6	49.5^**^
	30–39	5,407	16.5	2,866	53.0	50.3
	40–49	6,284	19.1	3,064	48.8	45.5
	50–59	8,313	25.3	3,489	42.0	41.4
	60–69	6,522	19.8	2,111	32.4	30.9
	≥70	4,068	12.4	867	21.3	18.1
Ethnicity	Han	24,805	75.5	7,745	31.2	36.8^**^
	Ethnic minorities	8,052	24.5	5,842	72.6	70.1
Education	Primary or below	11,988	36.5	4,002	33.4	31.3^**^
	Junior high school	12,727	38.7	5,952	46.8	49.0
	Senior high school	5,562	16.9	2,445	44.0	46.0
	Undergraduate or above	2,580	7.9	1,188	46.0	45.3
Marital status	Married	29,058	88.4	11,591	39.9	40.4^**^
	Not married	3,799	11.6	1996	52.5	52.3
Occupation	Famer	17,394	52.9	8,007	46.0	45.0^**^
	Employee	3,701	11.3	1784	48.2	51.7
	Student	1981	6.0	786	39.7	38.0
	Unemployed	9,781	29.8	3,010	30.8	36.3
Region	Urban	8,474	25.8	3,023	35.7	42.6
	Rural	24,383	74.2	10,564	43.3	42.9
In total		32,857	100.0	13,587	41.4	42.8

^**^p < 0.001; %^cd^, survey sample proportion; %^wtd^, adjustment of weighted proportion based on the 7th National Population Census Data of Hainan Province.

### Alcohol consumption patterns

#### Frequency of drinking

**Figure 2:**
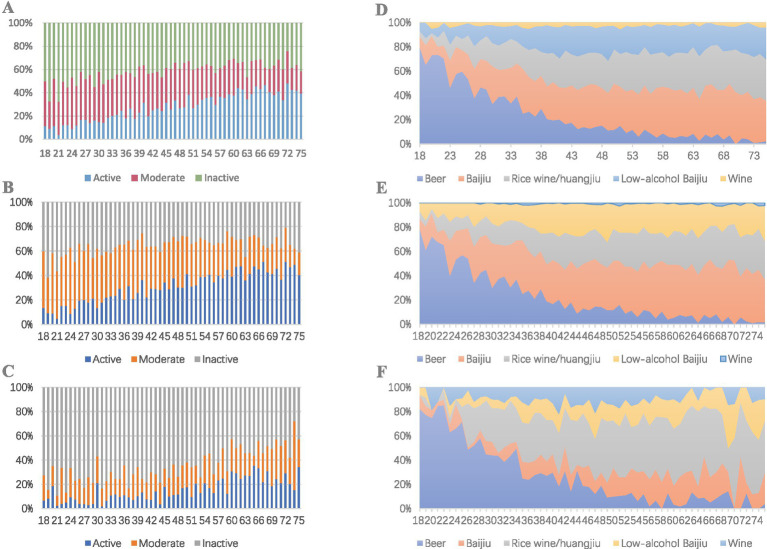
Alcohol consumption frequency and types by age group. **(A)** Overall population alcohol consumption frequency by age. **(B)** Male alcohol consumption frequency by age. **(C)** Female alcohol consumption frequency by age. **(D)**. Overall population alcohol types by age. **(E)** Male alcohol types by age. **(F)** Female alcohol types by age.

The distribution of overall drinking frequency shows that individuals consuming alcohol for ≥5 days per week form the smallest active drinking group at 24.4%. In contrast, those drinking for ≤3 days per month comprise the largest inactive group at 43.2%. Notably, the proportion of active male drinkers (27.7%) is significantly higher than that of female drinkers (11.7%). The active drinking rate increases with age, with more than 40% of individuals aged 60 and above classified as active drinkers. Meanwhile, the proportion of individuals drinking alcohol 1–4 days per week gradually decreases. However, the decline in inactive drinkers is relatively small, and this trend is more pronounced among male drinkers (Table 2; Figures 2A–C). Regarding educational background, individuals with elementary education or less have a lower overall drinking rate but a significantly higher active drinking proportion of 36.2%, compared to just 10.3% among those with a bachelor’s degree or higher. Similarly, among agricultural workers, the overall drinking rate is lower than that of office workers; however, the active drinking rate is markedly higher than that of non-agricultural groups, with students showing the lowest active drinking rate at 10.1%. The active drinking rate in rural populations is significantly higher than that in urban populations.

**Table 2:** Distribution characteristics of alcohol consumption frequency and types among adults in Hainan Province.

		Consumption frequency (%^wtd^)			Alcohol types (%^wtd^)					Average alcohol consumption (mL^a^)				
		Active	Moderate	Inactive	Beer	Baijiu	Low-alcohol Baijiu	Wine	Rice-wine/Huangjiu	Beer	Baijiu	Low-alcohol Baijiu	Wine	Rice-wine/Huangjiu
Gender	Male	27.7	36.2	36.0^**^	25.3	33.3	19.7	0.7	20.9^**^	487.0^**^	120.7^**^	157.1^**^	132.5^**^	159.8^**^
	Female	11.7	18.3	69.9	35.2	12.8	10.6	10.2	31.2	250.8	87.8	99.7	93.4	95.2
Age (years)	18–29	12.6	35.7	51.7^**^	56.3	19.0	10.0	2.3	12.5^**^	458.5^**^	128.8^**^	163.6^**^	122.2^**^	137.4^**^
	30–39	19.7	35.2	45.1	31.2	27.9	16.1	3.0	21.7	402.9	132.6	163.5	114.0	152.9
	40–49	27.3	33.1	39.6	15.4	31.7	23.3	3.0	26.6	342.5	122.6	159.4	84.6	146.7
	50–59	34.2	28.8	37.0	9.5	36.1	22.0	2.8	29.6	302.3	106.9	143.4	90.8	137.3
	60–69	40.9	24.0	35.0	6.0	38.7	21.2	1.9	32.2	171.6	94.8	114.0	86.7	115.3
	≥70	40.3	22.0	37.6	2.3	35.8	24.8	3.0	34.1	110.5	83.5	92.5	69.0	98.5
Ethnicity	Han	22.7	32.1	45.2^**^	30.6	31.5	18.3	3.1	16.5^**^	390.4^**^	108.5^**^	137.4^**^	95.7^**^	130.1^**^
	Ethnic minorities	28.5	33.3	38.2	19.8	23.2	16.5	1.8	38.7	477.3	150.5	182.6	122.0	147.0
Education	Primary or below	36.2	30.8	33.0^**^	12.1	27.4	24.6	2.2	33.7^**^	282.8^**^	114.8^**^	159.1^**^	88.2^**^	137.9^**^
	Junior	25.8	35.2	39.0	25.7	29.2	17.7	2.2	25.2	426.7	122.7	148.2	99.6	139.6
	Senior	17.7	29.1	53.2	36.9	29.0	14.6	3.4	16.2	426.0	108.4	132.6	107.9	137.4
	Undergraduate	10.3	30.9	58.8	43.9	31.3	11.7	4.4	8.8	412.4	116.6	147.3	103.6	128.9
Marital status	Married	26.6	31.6	41.8^**^	21.1	31.2	19.7	2.9	25.1^**^	369.8^**^	112.8^**^	145.0^**^	96.4^**^	134.0^**^
	Not married	17.8	35.0	47.2	46.6	22.4	11.9	2.2	17.0	465.5	138.2	167.7	117.8	158.5
Occupation	Famer	33.4	32.5	34.1	13.3	29.6	22.5	1.4	33.1^**^	322.2^**^	122.2^**^	163.1^**^	111.3^**^	144.2^**^
	Employee	21.0	39.8	39.2	35.4	31.7	15.8	2.7	14.5	393.5	121.1	141.4	91.2	146.1
	Student	10.1	22.7	67.2	53.6	21.6	9.2	5.4	10.1	443.5	108.3	97.3	122.2	121.1
	Unemployed	14.5	30.4	55.2	39.6	28.6	13.1	4.3	14.4	466.4	107.3	126.1	90.1	113.1
Region	Urban	20.2	36.7	43.1^**^	30.2	20.4	19.8	3.5	26.1^**^	451.9^*^	112.3	167.6^*^	103.2	135.1
	Rural	25.9	30.9	43.2	26.4	32.2	17.0	2.4	21.9	389.9	118.4	141.0	98.9	139.6
In total		24.4	32.5	43.2	27.4	29.0	17.8	2.7	23.1	407.4	117.2	148.5	100.4	138.2

^*^p < 0.05; ^**^p < 0.001; %^wtd^, weighted proportion; active, ≥5 days/week; moderate, 1–4 days/week; inactive, ≤3 days/month.

a The average alcohol consumption per time follows a skewed distribution. After applying the natural logarithm and subsequently reverting it, we calculate the mean alcohol consumption.

#### Types of alcohol consumed

In terms of alcohol types, distilled Baijiu and beer are the most commonly consumed beverages among males, with 33.3% choosing distilled Baijiu and 25.3% choosing beer. In contrast, females prefer beer and rice wine (Huangjiu), accounting for 35.2 and 31.2%, respectively. Overall, beer is the most consumed alcoholic beverage among individuals under 30, with a consumption rate of 56.3%. However, as age increases, the consumption rates of distilled Baijiu and rice wine (Huangjiu) significantly rise, while beer consumption declines markedly. Among males, there is a pronounced increase in the consumption of both distilled and low-alcohol Baijiu. In contrast, females exhibit a significant rise in the consumption of rice wine (Huangjiu), with a notably higher consumption rate compared to males (Table 2; Figures 2D–F).

#### Characteristics of average alcohol consumption per time

**Figure 3:**
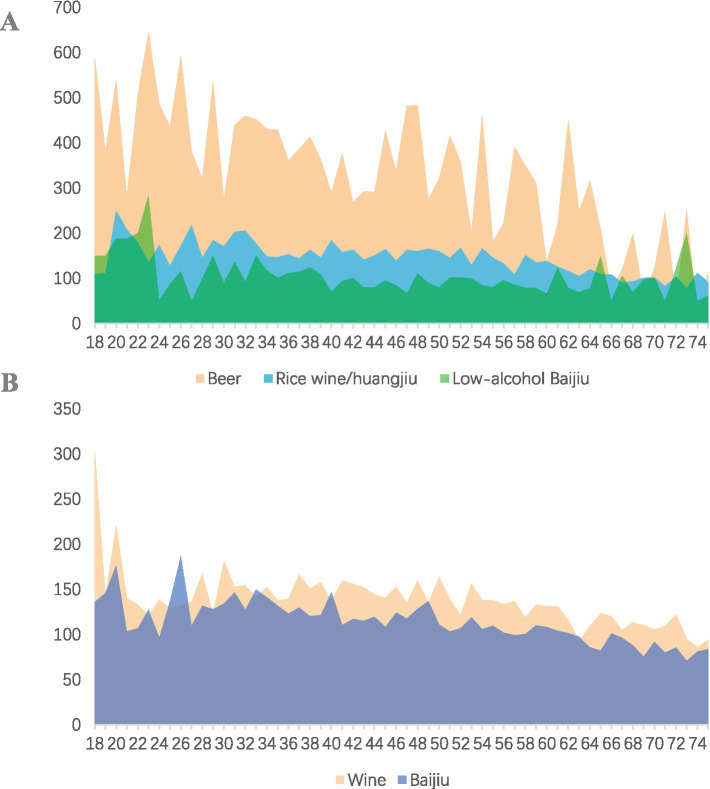
Average alcohol consumption per time by age. **(A)** Average consumption of beer, low-alcohol baijiu and wine. **(B)** Average consumption of Huangjiu and Baijiu.

Males have the highest average beer consumption per time at 487.0 mL, while females average 250.8 mL. Average consumption of other types of alcohol is relatively similar across genders. Notably, beer consumption declines significantly with age, while the decrease in the consumption of other types of alcohol is less pronounced. Ethnic minorities exhibit higher average consumption of all alcohol types compared to the Han population. Individuals with an education level of elementary school or below have significantly lower average beer consumption than those with higher education levels; however, they have the highest average consumption of low-alcohol Baijiu at 159.1 mL. In rural populations, both farmers and workers predominantly consume low-alcohol Baijiu in addition to beer (Table 2; Figure 3).

### Alcohol intake and multivariate analysis

**Figure 4:**
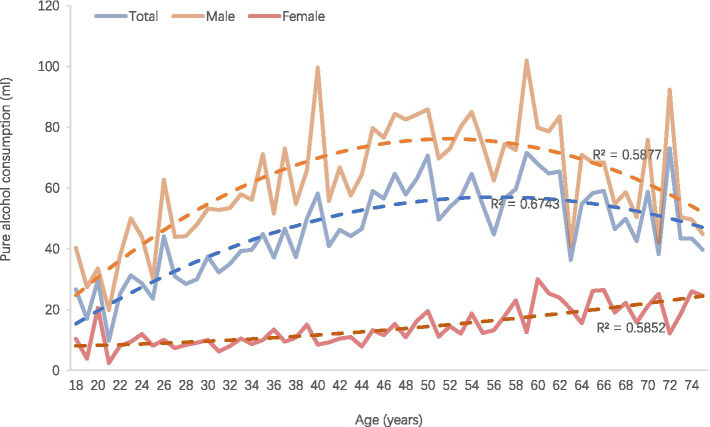
Weekly average pure alcohol intake.

Table 3 displays the distribution of weekly alcohol intake among adults in Hainan Province. The results indicate that the average weekly alcohol consumption for men is 59.8 mL, with 43.6% consuming over 100 mL. In contrast, women have an average intake of 10.9 mL, with 67.0% consuming ≤20 mL. Overall, alcohol consumption significantly increases with age, peaking at age 60 before declining. However, women’s alcohol intake continues to rise with age (Table 3; Figure 4). Regarding other demographic characteristics, ethnic minorities, individuals with lower educational attainment, married individuals, farmers, and rural populations exhibit significantly higher weekly alcohol intake. Notably, those with an education level of elementary school or below have consumption rates exceeding 62 mL per week, with over 47% consuming more than 100 mL per week. Further, we found all the unmarried male employees aged 60–69 years with primary or below education consumed over 100 mL pure alcohol per week, as well as those unmarried male employees aged 50–59 years with senior education. In the meantime, 91.5% unmarried females farmers of Han population with junior school education were found consuming over 100 mL pure alcohol.

**Table 3:** Weekly alcohol consumption among adults in Hainan Province.

		Weekly average pure alcohol intake, mL^a^		Weekly average pure alcohol intake, %^wtd^				
		$\bar{x}\pm S$	*β* ^std^	≤20 mL	(21–100)mL	>100 mL	OR^b^ (95%CI)	OR^c^ (95%CI)
Gender	Male	59.8 ± 5.5^**^	0.40	27.4	29.1	43.6	4.10 (4.06–4.13)	12.17 (12.06–12.28)
	Female	10.9 ± 5.8		67.0	20.3	12.7	Ref.	Ref.
Age (years)	18–29	26.7 ± 6.4^**^	0.05	44.8	28.6	26.6	1.19 (1.17–1.21)	0.80 (0.79–0.81)
	30–39	39.5 ± 6.4		36.3	28.6	35.2	1.20 (1.18–1.22)	0.91 (0.90–0.93)
	40–49	53.4 ± 6.1		30.2	28.5	41.3	1.32 (1.30–1.34)	1.07 (1.06–1.09)
	50–59	58.2 ± 5.9		30.5	24.3	45.2	1.11 (1.09–1.13)	1.14 (1.12–1.16)
	60–69	55.0 ± 6.0		30.7	23.6	45.7	1.17 (1.14–1.19)	1.35 (1.32–1.37)
	≥70	44.9 ± 5.4		35.4	22.8	41.7	Ref.	Ref.
Ethnicity	Han	37.8 ± 6.2^**^	0.14	37.5	27.8	34.6	0.60 (0.59–0.60)	0.44 (0.44–0.44)
	Ethnic minorities	54.1 ± 6.5		31.1	25.7	43.1	Ref.	Ref.
Education	Primary and below	62.4 ± 6.4^**^	0.10	28.7	23.0	48.3	1.44 (1.42–1.46)	2.14 (2.12–2.16)
	Junior	48.3 ± 6.1		32.0	28.3	39.7	1.41 (1.39–1.42)	1.57 (1.56–1.59)
	Senior	29.2 ± 6.1		42.8	29.1	28.1	1.12 (1.11–1.13)	0.99 (0.98–1.00)
	Undergraduate	23.6 ± 5.7		49.0	27.6	23.5	Ref.	Ref.
Marital status	Married	44.5 ± 6.2^**^	0.03	34.4	27.1	38.5	1.21 (1.20–1.22)	1.17 (1.16–1.18)
	Not married	35.1 ± 6.4		39.6	27.5	32.9	Ref.	Ref.
Occupation	Famer	62.2 ± 6.3^**^	0.11	28.5	24.3	47.2	1.12 (1.12–1.13)	1.90 (1.88–1.91)
	Employee	43.1 ± 5.5		31.9	33.3	34.7	1.47 (1.46–1.49)	1.61 (1.60–1.63)
	Student	17.1 ± 6.1		54.5	27.1	18.4	1.03 (1.01–1.04)	0.87 (0.86–0.88)
	Unemployed	26.3 ± 5.8		46.0	28.4	25.6	Ref.	Ref.
Region	Urban	38.7 ± 5.6^**^	—	37.3	29.9	32.7	1.06 (1.05–1.06)	0.88 (0.87–0.89)
	Rural	43.3 ± 6.6		35.0	26.2	38.8	Ref.	Ref.
In total		42.0 ± 6.3		35.7	27.2	37.1	-	-

^**^p < 0.001; %^wtd^, Weighted proportion; β^std^, the standardized β.

a The weekly alcohol intake exhibits a skewed distribution. After applying the natural logarithm transformation, the mean consumption is calculated by reversing the natural logarithm.

b Multivariable logistic regression was conducted with ≤ 20 mL as the reference value, yielding the odds ratio (OR) for the (21–100) ml category. Additionally, for > 100 mL, the OR was also derived using ≤ 20 mL as the reference.

Multiple linear stepwise regression results indicate that gender is the most significant demographic factor associated with weekly alcohol intake, with a standardized *β* value of 0.40, and ethnicity was with a standardized β of 0.14. Multivariable logistic regression analysis shows that men consuming between 21 and 100 mL weekly have a risk of 4.10 (4.06–4.13) times that of women, compared to those consuming ≤20 mL. Men consuming over 100 mL weekly have a risk of 12.17 (12.06–12.28) times that of women. Additionally, individuals with an education level of elementary school or below have an odds ratio (OR) of 1.44 (1.42–1.46) for consuming 21–100 mL weekly compared to those with a bachelor’s degree. They also have an OR of 2.14 (2.12–2.16) for consuming over 100 mL weekly.

## Discussion

This study conducted a comprehensive epidemiological survey and analysis of drinking patterns and alcohol consumption among adults in Hainan Province. It revealed significant differences in drinking behaviors and alcohol intake based on various demographic characteristics. The findings indicate that sociodemographic factors—such as gender, age, ethnicity, education level, marital status, occupation, and region—are closely linked to drinking behavior. This provides essential scientific evidence for formulating public health policies and intervention strategies.

The overall drinking rate among adults in Hainan Province is 42.8%, with men exhibiting a significantly higher rate (64.4%) compared to women (18.9%). This discrepancy suggests a persistent influence of gender norms and cultural customs on drinking behaviors. It also might be a result of the cultural and social context, that drinking is often considered as a part of social activities among male and the traditional tolerant attitude toward male drinking has also contributed to the increase in male drinking rates (10, 11). Studies have shown that men are more likely to relieve stress and anxiety by drinking (12). In addition, men tend to seek psychological comfort through drinking alcohol when facing life and work pressures (13, 14). The World Health Organization (WHO) reports that the global male drinking rate is approximately four times that of females (8, 9). Men’s drinking rates increase rapidly from age 18, peaking between 30 and 39 years. This indicates that early intervention is crucial before age 40. In contrast, women’s drinking rates peak early at ages 18 to 20, followed by a rapid decline. This highlights the need for early interventions targeted at female drinkers (9, 15). Previous research indicates that excessive drinking during youth can result in long-term health issues, underscoring the importance of addressing drinking patterns in young populations (15). Furthermore, as age increases, the proportion of active male drinkers rises, accompanied by a shift from beer to stronger spirits. This indicates a heightened risk (16). Meanwhile, women primarily consume rice wine and yellow wine, suggesting that interventions should account for age and gender-specific drinking preferences.

The drinking rate among ethnic minorities is 70.1%, much higher than the Han population’s 36.8%. Compared to Han nationality, drinking culture plays a more traditional role among ethnic minorities (10). Drinking is often treated as a racial cognition and ceremony in their daily life (17). This suggests that ethnic factors significantly influence drinking behaviors (18). This difference may stem from cultural traditions, social environments, or differing health education levels, highlighting the need for further investigation and culturally appropriate interventions (19). The study found that education level significantly affects drinking behavior. Individuals with junior high education or less have the lowest overall drinking rate (31.3%) but a higher proportion of active drinkers. This aligns with Jernigan’s findings that marginalized groups face greater alcohol-related health issues (20). This underscores the need for tailored educational interventions to reduce heavy drinking risks across different educational backgrounds. Although urban (42.6%) and rural (42.9%) residents have similar overall drinking rates, the higher active drinking rate in rural areas indicates a need for targeted public health messaging on drinking frequency and quantity (16, 21, 22).

The average weekly pure alcohol intake among adult men in Hainan Province is about six times that of women, aligning with global trends (8). Additionally, 43.6% of men consume over 100 mL of alcohol weekly. Multivariable regression analysis shows that men are more likely to engage in excessive drinking than women. Men’s weekly alcohol consumption gradually increases with age, peaking at 60 before declining, while women’s consumption keeps rising. The percentage of older adults consuming over 100 mL of alcohol weekly also rises, peaking at ages 60–69. This suggests that interventions for older populations should focus on reducing alcohol intake.

Individuals with primary education or less, along with agricultural workers, show lower overall drinking rates. However, their high levels of active drinking, preferred alcohol types, and average consumption reflect risky behaviors, with pure alcohol intake exceeding 62 mL. Previous studies show that health risks linked to alcohol consumption are significantly higher in lower socioeconomic groups. This suggests that low-education and agricultural populations in Hainan are at elevated risk for alcohol-related health issues and need targeted intervention. Notably, students have much lower drinking rates, consumption levels, and frequency compared to other groups. This indicates that improved health education for students could significantly enhance intervention effectiveness (23).

Alcohol drinking is suggested as a social psychological demand, including a need for interpersonal relationship, less empathy ability, high stress, depression, or coping mechanisms, which are key drivers of alcohol use and drinking patterns (24–27). As is concluded, the level of empathy was significantly lower in the group of alcohol-dependent population than others, in whom alcohol use might be a compensation for their intrinsic weakness (26). Though we did not include such psychological aspects into analysis, our results offered similar clues. Groups experiencing high drinking rate focused on males, youngsters, ethnic minorities or enterprise employees, that were indicated bearing heavy social stress or emotional weakness. This is in line with several former researches indicating alcohol misuse increases the risk of subsequent behavioral disorders (14).

The study bears several limitations. First, the cross-sectional design restricts the ability to establish causal relationships between sociodemographic factors and drinking behaviors, necessitating longitudinal research for deeper insights. Additionally, reliance on self-reported data may introduce bias. Moreover, while the study recognizes ethnic and cultural influences on drinking behaviors, it does not explore the specific cultural contexts shaping these patterns, indicating a need for further qualitative research. Also, we did not acquire globally validated tools like Alcohol Use Disorders Identification Test (AUDIT), to evaluate drinking patterns or alcohol consumption, which might make our results difficult in external validity across regions. The focus on adults in Hainan Province may limit the generalizability of the findings to other regions with different cultural or economic backgrounds, and a more detailed analysis within specific age cohorts is warranted to understand generational differences in drinking patterns.

## Conclusion

This study highlights the complex and diverse drinking behaviors in Hainan Province. By identifying high-risk groups based on gender, ethnicity, education, and occupation, the study offers clear guidance for public health strategies. Future efforts should emphasize culturally sensitive and targeted interventions, especially aimed at reducing drinking frequency and quantity among vulnerable populations to minimize alcohol-related health risks.

## Data Availability

The raw data supporting the conclusions of this article will be made available, upon reasonable request to the corresponding author.
